# Host Infection beyond the Traditional Range of Sclerotium (Athelia) rolfsii with Physalis minima

**DOI:** 10.6026/97320630013333

**Published:** 2017-10-31

**Authors:** Subhadip Nandi, Satyajit Hembaram, Arindam Adhikari, Basant Kumar Tiwari, Subrata Dutta

**Affiliations:** 1 Department of Plant Protection, PSB, Visva-Bharati, Sreeniketan, Birbhum, West Bengal, India; 2 AICRP on Vegetable crops, Directorate of Research, B.C.K.V, Kalyani, Nadia, West Bengal, India; 3 Centre for Bioinformatics, Pondicherry University, Pondicherry-605 014, India

**Keywords:** Physalis minima, Sclerotium rolfsii, Internal Transcribed Spacer region, Molecular clock, Site specific evolutionary rate, KC543584

## Abstract

Physalis minima is an herbaceous plant and inhabitant of the porous and organic matter containing soil of bunds in crop fields, wastelands, around the houses, and on the roadsides. S. rolfsii is soil borne and it can infect over 500 plant species of different families. It is of interest to study the pathogenesis of S. rolfsii on P. minima. The S. rolfsii isolated from P. minima (physr1) was characterized by morphology and sequence of Internal Transcribed Spacer (ITS) region. The population structure determination and phylogenetic analysis showed the isolate physr1 significantly differs from other isolates. The null hypothesis of equal evolutionary rate was rejected throughout the Maximum likelihood (ML) tree topology of different S. rolfsii ITS sequences. The site-specific mean (relative) evolutionary rate analysis showed that most of the sites (80.59 % sites) evolved at a slower rate than average. Finally, the result of Tajima's neutrality test indicated that the population of S. rolfsii has recently begun to expand and that's why the pathogen was infecting the new host P. minima and pose a serious threat of infecting several other cropped and non-cropped hosts.

## Background

Physalis minima L. (Ground cherry plant - local name: ban tipariya - Bengal) is an herbaceous plant that belongs to the family solanaceae, and is commonly found on the bunds of the crop fields, wastelands, around the houses, on roadsides, etc., where the soil is porous and rich in organic matter. It is an annual herbaceous plant having a very delicate stem and leaves and a common weeds in arable and non-cultivable lands. The plant has been used as a diuretic for various urinary problems and to relieve pain too (analgesic action). It is an important weed for some commercially important crop plants. S. rolfsii, the polyphagous fungus has a wide host range of 500 species in about 100 families including groundnut, green bean, lima bean, onion, garden bean, pepper, potato, sweet potato, tomato and water melon worldwide causing huge losses [1]. Though the fungus is seed and soil borne, soil borne inoculum is more important in causing infection and disease development. The fungus S. rolfsii produces abundant white fluffy, branched, septate mycelium with clamp connections only on the main hyphae, which spread like a fan. Small white tufts were formed on mycelium which later gives rise to smooth, hard and dark brown sclerotia. Sclerotia may be spherical or irregular in shape and at maturity resemble the mustard seed [2]. In addition to systematic studies, molecular data have been used in conjunction with independent criteria such as pathogenicity, host range, geographic origin, and vegetative compatibility groups (VCGs) to examine the diversity among and within populations of the higher fungal groups, including Ascomycetes, Basiodiomycetes and Fungi Imperfecti [3]. The nuclear ribosomal cistron is considered to be an ideal region for taxonomic and phylogenetic studies in a wide range of organisms including plants, animals and fungi [4]. Each rDNA unit consists of three ribosomal coding regions (18S, 5.8S, 26S) separated by intragenic spacers referred to as internal transcribed spacers (ITS). The ITS spacers (ITS I and ITS II) flank the 5.8S gene and are eliminated after transcription. In this present work we have investigated the novelty of this isolate with other S. rolfsii isolated from different hosts on the basis of morphological, ITS sequence and studied the impact of population structure on infection of S. rolfsii to a new host.

## Methodology

### Isolation and maintenance of S. rolfsii

The fungus was isolated on Potato Dextrose Agar (PDA) directly from surface sterilized diseased tissue and these were maintained on PDA (Potato-200 g; Dextrose-20 g; Agar-18 g; D/W 1000 ml) slants at 4°C [5]. Morphological characteristic of the fungus was observed through microscopic methods and cultural characteristics were recorded by growing the fungus on PDA plates at 28±1°C. Pathogenicity was tested using placing a parafilm-wrapped PDA plug bearing both mycelium and sclerotia near the collar region of healthy ground cherry plants. After 7 days, yellowing of basal leaves, followed by drooping of leaves and wilting was observed on inoculated plants. The noninoculated control plants, on which only PDA plugs were deposited, remained healthy. The fungus was re-isolated from inoculated plants. Mycelial vegetative compatibility studies were done as standard method [6].

### DNA extraction from S. rolfsii

Discs of 5 mm diameter S. rolfsii isolates were cut from periphery of an actively growing 7 days old culture on PDA and inoculated into 250 ml conical flask containing 50 ml of sterile potato dextrose broth. The flasks were kept in static mode at 28±2°C (mrc incubator). The resultant growth of mycelial mat was harvested and excess moisture was completely removed through sterile blotting paper and used for DNA extraction. The total genomic DNA of S. rolfsii isolate (physr1) was extracted from vegetative mycelium as standard method [7]. The quality and quantity of DNA was analyzed by running 2 μL of each sample mixed with 2 μl of 10x loading dye in 1% agarose gel. The DNA from all isolates produced clear sharp bands in one per cent agarose gel indicating the good quality of DNA. The DNA has been quantified by comparing with the 1 kb size marker (Genei, Bangalore) and by spectrophotometer (Jasco V630).

### DNA sequencing and BLAST analysis

PCR amplification of Internal Transcribed Spacers (ITS) region of rDNA was performed using universal primers ITS-1 as forward primer and ITS-4 as reverse primer [8] in Biorad MyCycler. Direct sequencing of PCR products was conducted for positive strand in forward direction using Applied Biosysterns sequencers 373A and 377. The sequence reaction was conducted using the PRISM Dye Terminator Cycle Sequencing kit (Applied Biosystems, Foster City) following the manufacturer's instructions. The trimmed nucleotide sequence was first locally aligned using Basic Local Alignment Search Tool (BLAST) algorithm provided by National Centre for Biotechnology Information (NCBI) with nonredundant nucleotide data base in default parameters set up. The similar sequences were chosen on the basis of query coverage, maximum identity and expect value (e-value) for identification of query sequence. ITS sequences of S. rolfsii isolated from various hosts were retrieved for further phylogenetic analysis (Table 1). The various nucleotide properties of physr1 were calculated on MEGA 5.0 [9].

### DNA sequence analysis for evolutionary studies

The sequences for the ITS region of S. rolfsii separately aligned using ClustalW 1.6 [10] integrated in software MEGA5.0, using default parameters. To identify the most appropriate nucleotide substitution model for the ITS sequences, an algorithm was used which is implemented in MEGA 5.0. The chosen model was then used to obtain the maximum likelihood (ML) tree [11] with 1000 bootstrap replicates and the distance matrix [12] of genetic divergence among sequences. Both analyses were conducted in MEGA 5.0. The molecular clock hypothesis [12] was tested with the likelihood ratio test, by comparing the likelihood values of the given ML tree topology with and without assuming a molecular clock. The mean (relative) evolutionary rate for each site of nucleotide was estimated under Tamura (1992) model [12] with invariant site (+I) as this model has second lower Bayesian Information Criterion (BIC) value just higher than Tamura (1992) model [12] with uniform rate. There were total 510 positions in final data set. This final data set was subjected to Tajima's Neutrality Test [13] to evaluate the neutral theory of molecular evolution and to identify the population structure. The estimate of selection pressure on each codon was not undertaken as the ITS region is non-coding part of fungal genome. The ITS sequence of the S. rolfsii (Athelia rolfsii) isolated from P. minima submitted to NCBI nucleotide sequence database (Accession No KC543584).

## Result and Discussion

**Figure 1:**
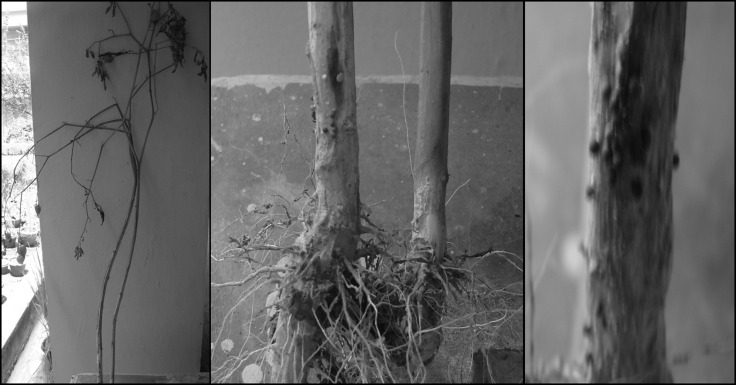
Sclerotium rolfsii infected Physalis plant

**Figure 2:**
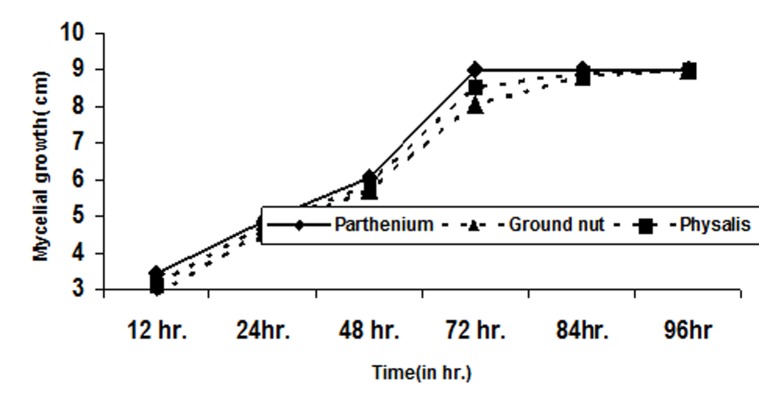
Mycelial growth of different S. rolfsii isolate

**Figure 3:**
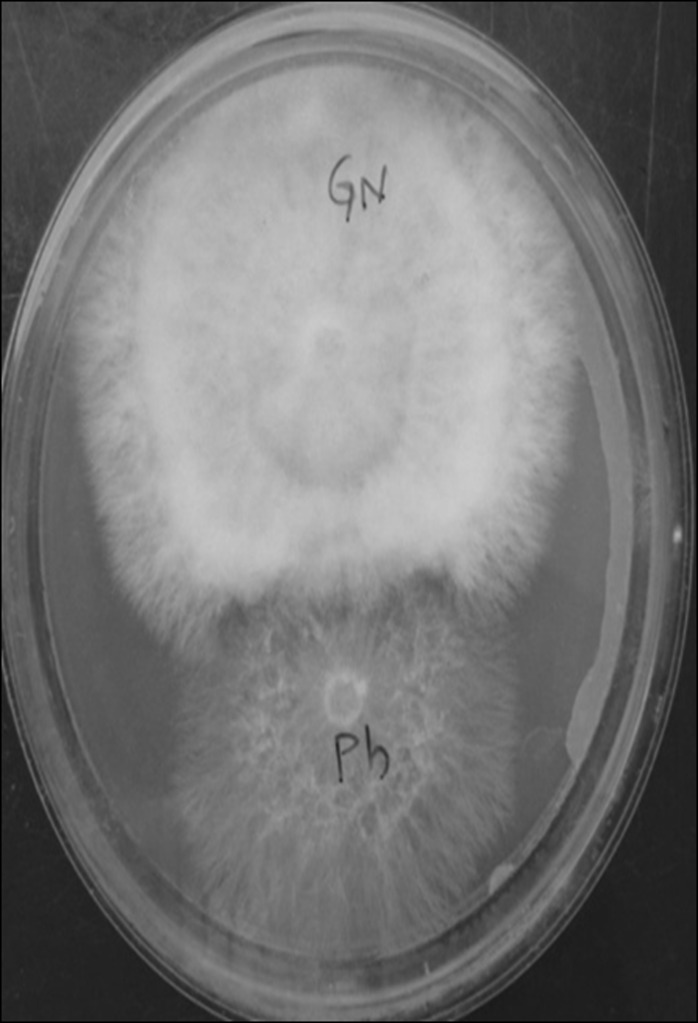
Compatibility study of different S. rolfsii isolates

In September 2010, a severe basal rot was observed on P. minima in C-Block Farm, Bidhan Chandra Krishi Viswavidyalaya, Nadia (West Bengal, India). Symptoms start with yellowing and drooping of leaves, with wilting of plants and a thin white cottony mycelial growth was observed at the collar region of the plant. On the diseased areas, small brown spherical sclerotia were observed, associated with the rotting of the tissues (Figure 1). Infected plants were taken from the field and isolations from field-grown plant yielded S. rolfsii that was identified on the basis of morphology. S. rolfsii is a cosmopolitan pathogen of many cultivated crops and weeds [14]. On the basis of morphological studies it was recorded that the fungus having white branched hyphae of 4.37 μm (3.0 - 6.25 μm) diameter, with clamp connections. Sclerotia smooth, spherical to ellipsoidal, light brown becoming dark brown with age and 159.48 μm (134.86 - 200.46 μm) in diameter. The morphological and cultural characteristics of physr1 were compared with Parthenium and groundnut isolates. The groundnut and Parthenium isolates of S. rolfsii produced sclerotia at 72 and 84 hrs after inoculation of PDA media whereas, Physalis isolate developed the sclerotia at 120 hrs after inoculation. The result depicted in Table 2 is the highest time required for the formation of sclerotia in Physalis isolates of S. rolfsii as compared to other isolates. The average size of the sclerotia of Physalis isolates (159.48 μm) was much lesser than that of groundnut isolates (286.03 μm). The growth rate in PDA medium of Parthenium isolate was faster as compared to groundnut and Physalis isolates (Figure 2). On the basis of compatibility study, it was also observed that Physalis isolates was not compatible with groundnut and Parthenium isolates with varied degrees of non-compatibility (Table 3 & Figure 3).

**Figure 4:**
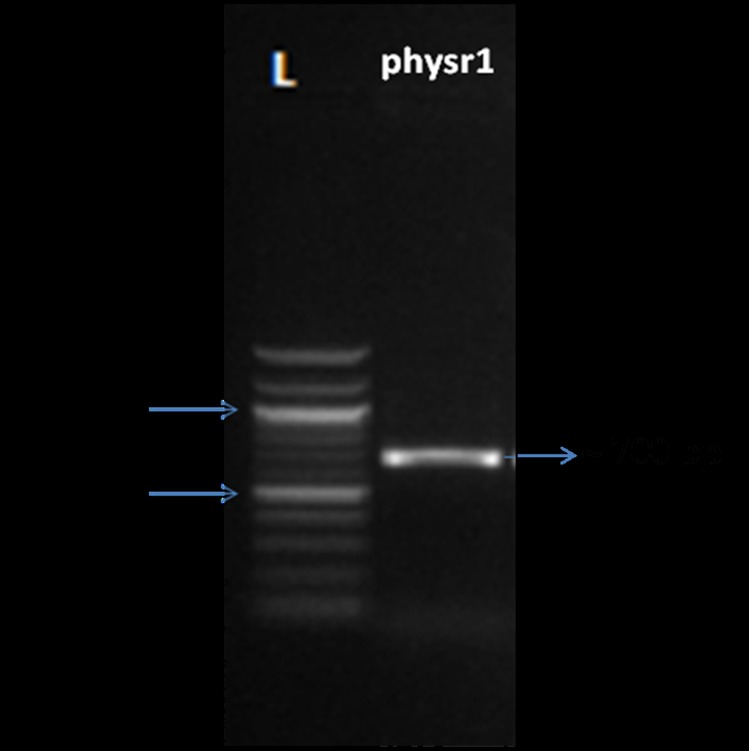
Agarose gel (1.5%) electrophoresis of amplified ITS product of S. rolfsii

**Figure 5:**
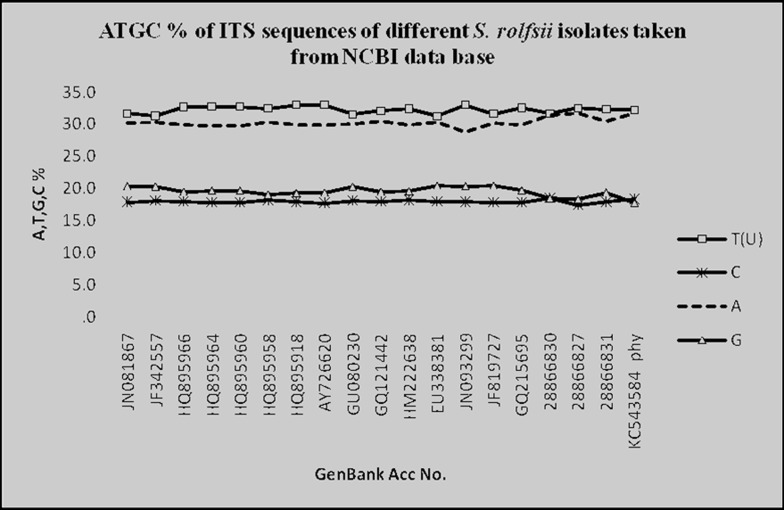
Nucleotide composition of ITS sequences of different S. rolfsii isolates

**Figure 6:**
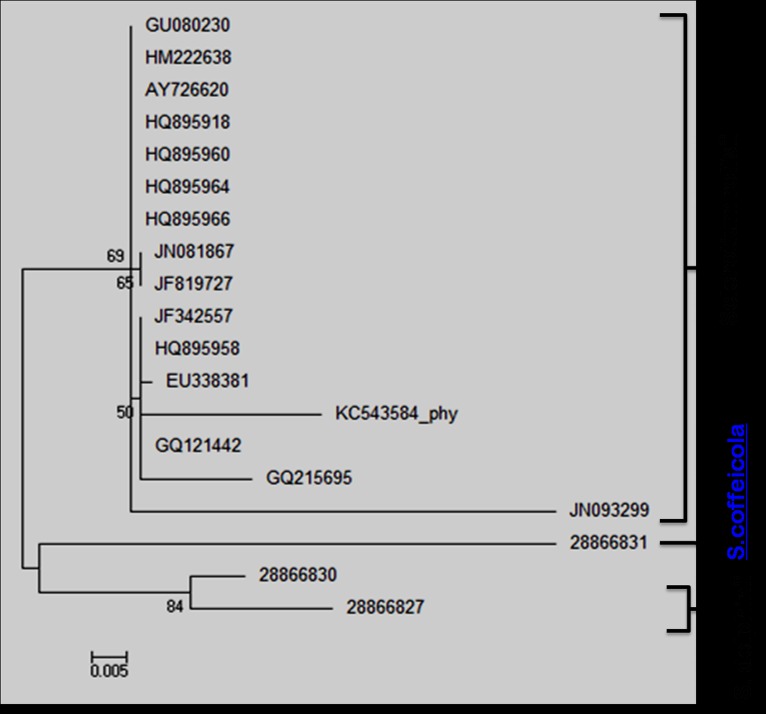
Phylogenetic tree of Sclerotium sp. on the basis of Internal Transcribed Spacer sequences.

**Figure 7:**
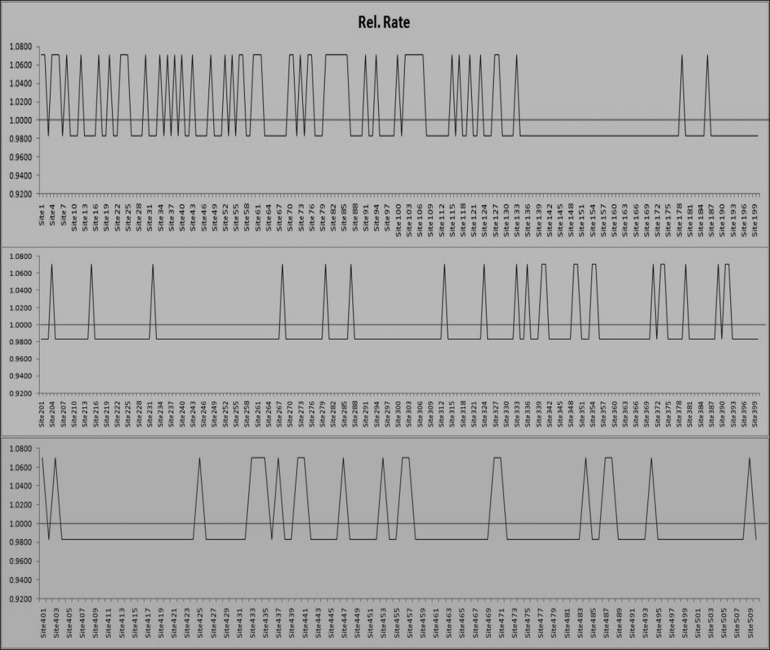
Mean (relative) evolutionary rate for each site of nucleotide subjected to analysis. There were a total of 510 positions in the final dataset

Gel electrophoresis of PCR product amplified by primers ITS1 and ITS4 from physr1 isolate yielded a uniform band of approximately 700 bp (Figure 4). Thus it was identified that the strain physr1 was Sclerotium rolfsii (Perfect stage: Athelia rolfsii) [15]. The BLAST result of ITS sequence showed that the isolate had 97% sequence similarity (score=1018, Expect value=0.0, Query coverage = 100%) with the same isolate Athelia rolfsii 1112 (JN241565). The sequences showed the domination of AT content in the ITS region of different S. rolfsii (Figure 5). The frequency of AT in different S. rolfsii ranged from 61.55% (JF342557) to 64.23% (28866827). The average AT content of all S. rolfsii retrieved from database showed 62.45% where the isolate physr1 contains 63.93%. Table 4 showed the evolutionary distance values between combinations of the ITS sequences in the various species of Sclerotium. A consensus ML tree was reconstructed from these aligned sequences (Figure 6). On the basis of ITS sequence homology three major clusters were formed with the distance in between two sequences ranged from 0.000 to 0.142. The sequence of an Indian S. rolfsii (JN093299) showed maximum distance (0.142) with S. coffeicola (28866831). The average distance in between the first group (contains sixteen S. rolfsii sequences) and second group (contains two S. delphinii sequecces) was 0.049 followed by 0.103 with the third group (contains one S. coffeicola sequence). Thus it can be clearly depicted that S. rolfsii is much more closely related with S. delphinii than S. coffeicola which supports the findings of previous researchers [16] who observed closed relationship in between S. rolfsii and S. delphinii on the basis of restriction maps of ITS regions only. The isolate physr1 exhibited highest similarity with S. rolfsii sequences JF342557, HQ895958, GQ121442 and GQ 215695 with the distance value of 0.028 where as mostly distant to S. coffeicola sequence (28866831) with the distance value of 0.125. The isolate physr1 exhibited the closer relationship with S. delphinii (Distance value 0.071) than S. coffeicola. The null hypothesis of equal evolutionary rate throughout the ML tree topology was rejected at a very high significance level (P <1.04 X 10-15). So, the alternative hypothesis i.e., 510 nucleotides showed different evolutionary rate was accepted. The mean (relative) evolutionary rates were scaled such that the average evolutionary rate across all sites was 1. This means that sites showing a rate < 1 are evolving slower than average and those with a rate > 1 are evolving faster than average. These relative rates were estimated under the Tamura (1992) model (+I). A discrete Gamma (+G) distribution was used to model evolutionary rate differences among sites. Some sites were allowed to stay invariant (+I). The estimate of the proportion of invariant sites was (6.5851% sites). It was clearly depicted in Figure 7 that the maximum sites showed a mean (relative) evolutionary rate < 1and thus it can be concluded that maximum sites (80.59 % sites) showed evolutionary rate slower than the average rate. Tajima's neutrality test clearly indicated that the nucleotide diversity (∏) among the population was very low (0.027096) and Tajima test statistic (D) was -2.125947. The result can be explained by two possibilities either the population size might be increasing or might have evidence for purifying selection at this locus. In purifying selection, mutations accumulate at silent sites but they were not likely to become very common in the population. As the population of S. rolfsii has recently begun to expand the mutations that occur were unlikely to be lost and the nucleotide diversity would be created slowly. It will create a new threat of S. rolfsii infection not only in crop plants but also in weeds. In weed host the pathogen can survive for long time and can serve as a source of primary infection site. In this concern, this is the right time for development of appropriate holistic disease management strategies against this gregarious pathogen.

## Conclusion

Thus, this is the first report of infection of S. rolfsii on P. minima and a study on the population structure of S. rolfsii globally. In this present work, we have showed the impact of population structure on expansion of host range of S. rolfsii. On the basis of cultural, morphological, compatibility and molecular Phylogenetic studies, it may be concluded that Physalis isolate of S. rolfsii is different from that of groundnut (isolated in our lab), Parthenium (isolated in our lab) and other hosts (sequences taken from NCBI data base).

## Figures and Tables

**Table 1:** List of the ITS sequences of Sclerotium sp.

S No	Accession no.	Location	Host	Organism
1	JN081867	China	Sweetpotato	A. rolfsii
2	JF342557	South Korea	Daucus carota var. sativa	A. rolfsii
3	HQ895966	Vietnam	groundnut	A. rolfsii
4	HQ895964	Vietnam	groundnut	A. rolfsii
5	HQ895960	Vietnam	groundnut	A. rolfsii
6	HQ895958	Vietnam	groundnut	A. rolfsii
7	HQ895918	Vietnam	groundnut	A. rolfsii
8	AY726620	Mexico	pepper	A. rolfsii
9	GU080230	Southern Spain	pepper	A. rolfsii
10	GQ121442	China	Zamioculcas zamiifolia	A. rolfsii
11	HM222638	Chile	Orobanche ramosa	A. rolfsii
12	EU338381	South Carolina	Bottle Gourd	A. rolfsii
13	JN093299	India	-	A. rolfsii
14	JF819727	China	peanut	A. rolfsii
15	GQ215695	India	banana	A. rolfsii
16	KC543584	India	Physalis minima	A. rolfsii
17	28866830	Japan	-	A. delphinii
18	28866827	Japan	-	A. delphinii
19	28866831	Japan	-	S.coffeicola

**Table 2:** Morphological characterastic of different Sclerotium isolates

Name of the host	Hyphae diameter (μM)	Hyphae colour	Branched or unbranched	Clamp connections	First sclerotia formation time (hr.)	Sclerotia texture	Sclerotia colour	Sclerotia diameter (μm)
Physalis	4.37 (±0.91)	White	Branched	Present	120	Smooth	Dark brown	159.48 (±20.88)
Pathenium	4.50 (±1.16)	White	Branched	Present	72	Smooth	Dark brown	138.97 (±13.65)
Ground nut	3.84 (±1.41)	White	Branched	Present	84	Smooth	Dark brown	286.03 (±58.00)

(Values in parentheses are standard deviation of mean value)								

**Table 3:** Compatibility study of different Sclerotium isolates

Name of the host	Physalis	Pathenium	Ground nut
Physalis	C	N	N
Pathenium	N	C	N
Ground nut	N	N	C
C = compatible; N = non-compatible			

**Table 4:** Distance matrix of DNA sequences computed on the basis of Tamura 3 parameter with uniform rate.

Sl	Acc. No.	1	2	3	4	5	6	7	8	9	10	11	12	13	14	15	16	17	18	19
1	JN081867	-																		
2	JF342557	0.002	-																	
3	HQ895966	0	0.002	-																
4	HQ895964	0	0.002	0	-															
5	HQ895960	0	0.002	0	0	-														
6	HQ895958	0.002	0	0.002	0.002	0.002	-													
7	HQ895918	0	0.002	0	0	0	0.002	-												
8	AY726620	0	0.002	0	0	0	0.002	0	-											
9	GU080230	0	0.002	0	0	0	0.002	0	0	-										
10	GQ121442	0.002	0	0.002	0.002	0.002	0	0.002	0.002	0.002	-									
11	HM222638	0	0.002	0	0	0	0.002	0	0	0	0.002	-								
12	EU338381	0.004	0.002	0.004	0.004	0.004	0.002	0.004	0.004	0.004	0.002	0.004	-							
13	JN093299	0.057	0.059	0.057	0.057	0.057	0.059	0.057	0.057	0.057	0.059	0.057	0.061	-						
14	JF819727	0	0.002	0	0	0	0.002	0	0	0	0.002	0	0.004	0.057	-					
15	GQ215695	0.002	0	0.002	0.002	0.002	0	0.002	0.002	0.002	0	0.002	0.002	0.059	0.002	-				
16	28866830	0.034	0.037	0.034	0.034	0.034	0.037	0.034	0.034	0.034	0.037	0.034	0.039	0.094	0.034	0.037	-			
17	28866827	0.052	0.054	0.052	0.052	0.052	0.054	0.052	0.052	0.052	0.054	0.052	0.056	0.113	0.052	0.054	0.032	-		
18	28866831	0.1	0.097	0.1	0.1	0.1	0.097	0.1	0.1	0.1	0.097	0.1	0.1	0.142	0.1	0.097	0.107	0.105	-	
19	KC543584_phy	0.03	0.028	0.03	0.03	0.03	0.028	0.03	0.03	0.03	0.028	0.03	0.03	0.09	0.03	0.028	0.062	0.08	0.125	-
